# Multisensor Monitoring System for Assessment of Locust Hazard Risk in the Lake Balkhash Drainage Basin

**DOI:** 10.1007/s00267-012-9950-2

**Published:** 2012-09-19

**Authors:** Pavel Propastin

**Affiliations:** 1 Cartograpphy, GIS and Remote Sensing, George August University Göttingen, Goldschmidt street 5, Göttingen, Lower Saxony Germany; 2 Institute of Bioclimatology, George August University Göttingen, Büsgen-weg 2, 37077 Göttingen, Lower Saxony Germany

**Keywords:** Locust risk, Lake Balkhash, River Ili, Water table, Vegetation dynamics, Remote sensing

## Abstract

Satellite and ground-based data were combined in a monitoring system to quantify the link between climate conditions and the risk of locust infestations in the southern part of Lake Balkhash’s drainage basin in the Republic of Kazakhstan. In this monitoring system, the Normalized Difference Vegetation Index (NDVI), derived from the SPOT-VGT satellite, was used for mapping potential locust habitats and monitoring their area throughout 1998 to 2007. TOPEX/Poseidon and Jason 1 altimeter data were used to track the interannual dynamics of water level in Balkhash Lake. Climate conditions were represented by weather records for air temperature and precipitation during the same period. The classification procedure, based on an analysis of multitemporal dynamics of SPOT-VGT NDVI values observed by individual vegetation classes, generated annual areas of ten land-cover types, which were then categorized as areas with low, medium, and high risk for locust infestation. Statistical analyses showed significant influences of the climatic parameters and the Balkhash Lake hydrological regime on the spatial extend of annual areas of potential locust habitats. The results also indicate that the linkages between locust infestation risk and environmental factors are characterized by time lags. The expansion of locust risk areas are usually preceded by dry, hot years and lower water levels in Balkhash Lake when larger areas of reed grass are free from seasonal flooding. Years with such conditions are favourable for locust outbreaks due to expansion of the habitat areas suitable for locust oviposition and nymphal development. In contrast, years with higher water levels in Balkhash Lake and lower temperature decrease the potential locust habitat area.

## Introduction

**Fig. 1:**
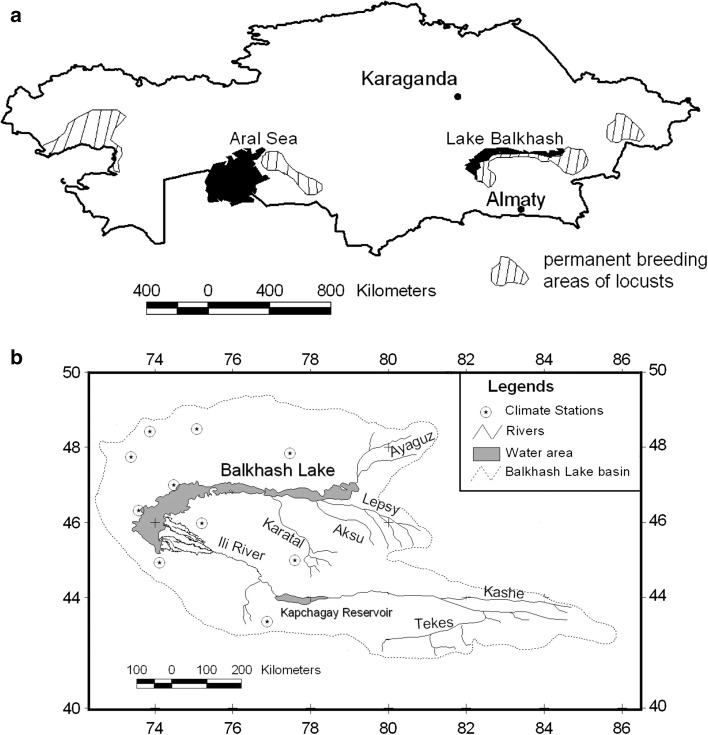
**a** Breeding areas of L. *migratoria migratoria* in Kazakhstan (modified from Tsyplenkov 1970). **b** Study area for prediction of locust infestation in the Lake Balkhash region

One of the most persistent and damaging natural hazards of Central Asia is locusts. With a periodicity of 2 to 10 years, swarms of locusts endanger agricultural production in the region by devastating crops and pastures (Tsyplenkov 1970; Kambulin 1992). Locusts also invaded settlements. For instance, one of the most damaging locust outbreaks occurred in July 1999, when swarms of locusts invaded the capital of Kazakhstan Astana, terrifying citizens and causing traffic accidents (Toleubayev and others 2007). The agent of the locust hazard in this large region is the Asian migratory locust (*Locusta migratoria migratoria)*, which is highly mobile and capable of devastating crops covering large areas. On appropriate surroundings, the species L. *migratoria migratoria* is able to multiply quickly and decrease agricultural production over large regions (Pedgley 1981). In the formerly Soviet Central Asia, the largest permanent breeding areas of the Asian migratory locust are located in Uzbekistan (Latchininsky and others 2007) and in Kazakhstan (Kambulin 1992) (Fig. 1).

With respect to the Republic of Kazakhstan, the permanent breeding area in the drainage basin of Lake Balkhash in Southeast Kazakhstan most economically important with respect to damage incurred (Toleubayev and others 2007). The total area of the River Ili delta in the southern part of the Lake Balkhash is approximately 1 million ha, and > 0.3 million ha within this total area are susceptible to locust infestations (Tsyplenkov 1970). Under favourable ecological conditions, this area produces large swarms of the Asian migratory locust, which causes devastating damage to ecosystems in the region. With a periodicity of 2 to 10 years, swarms of the Asian migratory locust endanger agricultural production in the region (Kambulin 1992; Toleubayev and others 2007; Khasenov 2001). Commonly, the outbreaks occur after abnormally dry years, when larger areas in the River Ili delta are free from flooding water. Such areas represent suitable habitats for locust oviposition (Latchininsky and others 2007; Sivanpillai and others 2006).

Remote sensing is an effective technology for collecting information on vegetation and earth surface conditions at several scales. Modern monitoring of the locust hazard, based on remote-sensing techniques, enables mapping of locust habitats, postdamage assessment, and observation of locust population dynamics at the scales from local to national (Latchininsky & Sivanpillai 2010). Remote-sensing techniques have been applied for forecasting infestation by locusts and damage assessment in Africa (Voss & Dreiser 1997; Despland and others 2004; Tratalos & Cheke 2006; Ceccato and others 2007; Tappan and others 1991), Asia (Ji and others 2005; Ma and others 2005; Sivanpillai and others 2006; Tian and others 2008; Liu and others 2008) and Australia (Deveson and others 2005; Hunter and others 2008).

Fine spatial-resolution data (30 m) from Landsat MultiSpectral Scanner and Thematic Mapper (TM) sensors have been used for mapping locust habitats at a local or subregional scale (Sivanpillai and others 2006; Ma and others 2005; Voss & Dreiser 1997). Commonly, the result of such studies is a single map of potential locust habitats based on satellite data classification (Latchininsky and others 2007; Latchininsky and Sivanpillai 2010; Sivanpillai and others 2006). However, due to considerable temporal heterogeneity of rainfall in drylands, mapping of locust habitats in semidesert and desert environments without taking into account the intra-annual and interannual rainfall dynamics has had only limited success (Latchininsky and others 2007).

To overcome this constraint, multitemporal satellite data (data collected at different times) can be used for mapping land-cover using vegetation seasonal (within year) and interseasonal (across several years) change information (Friedl and others 2002; Fensholt 2004). Such studies generally demand moderate- and coarse-resolution data with high repetition time of data acquisition. Data sets with global coverage originating from a number of satellite systems, such as Advanced Very High Resolution Radiometer (AVHRR), Satellite Pure Observation d’Tierra (SPOT), and Moderate Resolution Imaging Spectroradiometer (MODIS), have become available to the remote-sensing community in recent years. A number of studies showed good suitability of coarse- and moderate-resolution data for mapping locust habitats and monitoring habitat conditions in various regions (Sivanpillai & Latchininsky 2007; Ji and others 2005).

Commonly, multitemporal mapping of locust habitats and damage assessment, satellite-derived vegetation indices (VIs) have been used for capturing phenological changes of different vegetation classes (Sivanpillai & Latchininsky 2007; Zha and others 2005). Among a large number of various satellite-based VIs, the Normalised Difference Vegetation Index (NDVI) is the most used product for such studies. The NDVI serves as a general indicator of vegetation greening and is derived from the red and near infrared portions of reflected radiation as follows (Tucker 1979):

1 $$ {\text{NDVI}} = \left( {{\text{NIR}} - {\text{R}}} \right)/\left( {{\text{NIR}} + {\text{R}}} \right) $$

where R is the red portion of the spectrum, and NIR is the near infrared portion of the spectrum. Due to the absorbance qualities of chlorophyll, this value tends to be high where there is a large amount of green vegetation and low where vegetation is sparse or dry. The NDVI has been proved to correlate strongly with such characteristics of vegetation canopy as biomass, fractional vegetation cover, leaf area index, and fraction of photosynthetically active radiation (Asrar and others 1984; Daughtry and others 1992.

Recently, NDVI data sets with global coverage are routinely produced from AVHRR, SPOT, and MODIS sensors. These data sets are freely available to the remote-sensing community. A number of studies showed suitability of the NDVI time-series for monitoring the dynamics of insect populations, mapping of locust habitats, and damage assessment (Tratalos & Cheke 2006; Thomson & Connor 2000; Despland and others 2004; Li and others 2005; Zha and others 2005). Currently, there are a number of routine and operational locust forecasting systems based on the incorporation of locust survey information and the monitoring of habitat conditions using remote-sensing and geographic information systems (GIS), which have already been established and promoted by the Food and Agricultural Organization (Magor & Pender 1997; Latchininsky & Sivanpillai 2010).

During last two decades, some progress has been achieved in the remote sensing–based monitoring of potential locust hazard in Central Asia (Latchininsky 2007; Latchininsky and others 2007; Sivanpillai and others 2006, 2009). However, considering the importance of this region to global change and global food security (Henebry 2009), the achieved progress is not enough to close the existing research gap. Monitoring of locust hazard and the assessment of regions after damage have great importance with respect to recent efforts toward mapping carbon sequestration in the drylands of Kazakhstan (Propastin and others 2012).

This study aimed to contribute to the current research of locust hazard in the largest country of Central Asia: Kazakhstan. The article introduces a satellite-based monitoring system for the assessment of locust infestation risk in the Balkhash Lake region in Kazakhstan. In doing so, this study is not unique: Examples of this type of research have been reported from various regions (*e.g.*, Hunter and others 2008; Tian and others 2008; Liu and others 2008) and also from the Balkhash Lake drainage basin (Sivanpillai and others 2006). Instead, using well-established approaches for remote sensing–based mapping of locust habitats using single-date (Sivanpillai and others 2006; Ma and others 2005) or multitemporal data (Sivanpillai & Latchininsky 2007), the present study strived to make a contribution to current literature through the introduction and testing of a novel monitoring technique. The novelty of the proposed monitoring system is that it uses data from diverse satellite sources, e.g., satellite-based multitemporal NDVI, and satellite altimetry data, which are combined with ground measurements of climatic variables. The developed monitoring system is employed for (1) mapping and interannual monitoring the spatial distribution of breeding areas of L. *migratoria migratoria* in the Balkhash Lake drainage basin; and (2) quantifying the impact of climate factors and hydrological regimen of Balkhash Lake on the interannual variability of potential locust habitats.

## Study Background

### Study Area and its Environments

Lake Balkhash, the world’s fifth largest isolated water reservoir, has a volume of approximately 90 km³ and a catchment area >0.5 million km² (Fig. 1). The lake is situated in the Balkhash-Alakol depression and has a length of 605 km and a width varying between 4 and 74 km. The Lake Balkhash basin is internally drained, with the major loss of inflow water being evaporation from the lake surface. The main contributors to the Balkhash Lake are the Ili (78.2 % of the total surface inflow), the Karatal (15.1 %), the Aksu (0.13 %), and the Lepsy rivers (5.4 %). Long-term periodic fluctuations in the Balkhash Lake water levels have been suggested to be conditioned primarily climatically (but also, to a smaller degree, anthropogenically) and demonstrate their intimate connection with components of its water regime, especially runoff from the Ili River (Petr 1992; Piven 1990; Djanalieva & Bogachev 1992).

The climate in the region is strongly continental, with low annual precipitation (< 180 mm) and severe daily and annual temperature variations. The Lake Balkhash basin ecosystems comprise several plentiful wetlands in the deltas of its inflows. Floodplains of these wetlands are covered by thickets of reed grass (species *Phragmates communis* and *Calamagrostis pseudophragmates)*. These ecosystems are very fragile and directly correlate with the Lake Balkhash inflow from contributing rivers as well as its water level (Petr 1992; Tlenbekov & Piven 1993; Propastin 2008).

The construction of the Kaptchagay dam in the upper part of Ili River in 1970 took place during the last downward fluctuation in flow and lead to the steepest decrease in the lake’s water level since the initiation of measurement. This additionally affected the fragile ecosystems of Balkhash Lake and its environments (Tlenbekov & Piven 1993; Petr & Mitrofanov 1998).

### Problem of Locust Hazard in the Study Area

Asian migratory locusts have four development stages: hatching, fledging, wing-growth instars, and nymph. L. *migratoria migratoria* feeds on approximately ten plant species with a preference for two reed grass species, *P. communis* and *C. pseudophragmates*, which dominate the vegetation communities in floodplains of the river delta in the Lake Balkhash drainage basin. For that reason, the developmental stages of the Asian migratory locust in the Balkhash Lake region strongly coincide with the growing period of reed grass in the river’s deltas. Because the interannual and seasonal vegetation dynamics in the Lake Balkhash region are strongly correlated with the discharge of the Lake Balkhash inflow rivers and water level (Propastin and others 2007; Propastin 2008), the developmental pattern and population dynamics of the Asian migratory locust are synchronized with the hydrological regimen of its breeding areas (Kambulin 1992; Sivanpillai and others 2006).

The region of the formerly Soviet Central Asia includes five republics—Kazakhstan, Kyrgyzstan, Uzbekistan, Turkmenistan, and Tajikistan—which obtained independence after the collapse of the Soviet Union in 1991. The collapse of the Soviet Union in 1991 brought enormous and rapid changes in the economies of the post-Soviet republics. The former state system of preventing and combating locust hazard has been devastated (Latchininsky 2007). Locust survey and antilocust treatments were decreased to a minimum. Since the early 1990 s, monitoring and control of the Asian migratory locust in the Balkhash Lake drainage basin have been implemented at very low levels, and almost no control was applied until 1999. Together with continuing drought, these factors led to a build-up of locust populations, which required extensive control operations during 2000–2003 (Khasenov 2001; Toleubayev and others 2007). From this background, the development of effective, low-cost systems for monitoring locust hazard risk in the formerly Soviet Central Asia is of great importance. However, there is a gap in the state-of-the-art research in this region (Toleubayev and others 2007).

## Data Used in the Study

### Satellite Data

#### NDVI

The VEGETATION (VGT) sensor, on board the SPOT-4 satellite, has four spectral bands, blue (430 470 nm), R (610–680 nm), NIR (780–890 nm), and short-wave infrared (1580 to 1750 nm), of which R and NIR are used to calculate the NDVI. The SPOT-VGT provides daily global images at 1-km spatial resolution. These data are archived and used to generate global 10-day synthesis NDVI maps. The compositing method for generating 10-day NDVI data is to select an observation with the maximum NDVI value within a 10-day period (Holben 1986). This way, nonvegetated features, such as clouds and shadows, originally presented in the raw data were significantly decreased. The standard 10-day composite data are freely available to the public (http://free.vgt.vito.be).

For this study, we acquired SPOT-VGT NDVI data for the growing season (April through October) from 1998 through 2004 over the study area. Remaining noisy pixels in the NDVI data set were removed by employing a spatio-temporal filter, which calculated a new value for each noisy pixel from values of neighbouring non-noisy pixels (Chen and others 2004).

#### TOPEX/Poseidon and Jason 1 Altimeter Data

Satellite altimetry observations provide a unique opportunity to monitor the ocean and sea evolution in real time, accurately, with high temporal resolution and at the global scale. Radar altimetry was initially developed for oceanographic purposes, but in recent years it has been used as a powerful tool for monitoring inland water sources.

The main product of altimetry missions is surface-level height data (in the case of studies on inland water it is lake-level height data). Two altimetry data sets have been widely exploited in oceanographic and hydrological studies: TOPEX/Poseidon (T/P) in its original orbit with a 9.915625-day repetition time, and Jason 1, the follow-up mission to T/P, which was launched in 2001. Similar to T/P, Jason 1’s orbital repeatability is every 9.915625 days with the same global coverage.

**Fig. 2:**
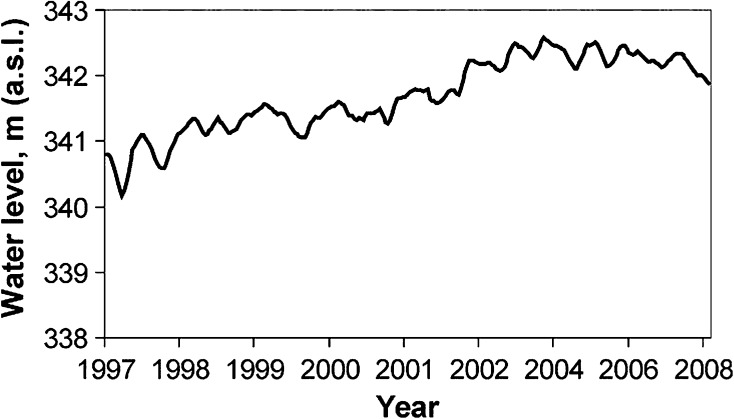
Ten-day oscillations of the water level in Balkhash Lake from 1997 through 2008 as calculated from T/P/Jason 1 data

For the present study, we obtained recorded variations of the water level in Lake Balkhash for the period 1993 through 2008 based on the T/P 16-year mean level (meters), which are available to public (http://www.pecad.fas.usda.gov/cropexplorer/global_reservoir/gr_regional_chart.cfmh). These data were then used to calculate variations in the water level of Lake Balkhash for the study period. The reliability of the satellite-derived estimates for lake level is discussed in details by Propastin (2012). In brief, the T/P-based estimates correlate strongly with the available data from level gauges (*R*² = 0.92, RMSE = 5.2 cm). The results of the altimetry-derived estimates for 1997 through 2008 are presented in Fig. 2.

### Climate Data

Ten-day records of air temperature and precipitation for 1998 through 2007 at 10 weather stations located in the study area (Fig. 1b) were used for the analysis of climatic conditions in the Balkhash Lake drainage basin. From these data, we calculated mean growing season temperature and average annual precipitation for the study region.

#### Ground Truth and Verification Data

Ground surveys were performed during our trips to the study area in summer 2004 to summer 2008 to collect field data for image classification and accuracy assessment. Altogether 120 locations within different vegetation-cover classes were collected using a GPS receiver (Garmin Ltd, Olathe, KS). The accuracy of geolocation measurements with the GPS devise was $\pm$5–8 m. In addition, information about changes in land cover was obtained through interviews with local authorities and specialists. We also analysed topographic maps at the scale 1:100,000 and fine-resolution Landsat TM (path 151–152, row 028–029, acquisition date July 15 1994, September 20 1992) and ETM+ (path 151–152, row 028–029, acquisition date May 23 2006, June 05 2006, August 02 2001) images with respect to the distribution of land-cover classes in the study area.

## Methodology

### Monitoring System for Locust Infestation Risk

**Fig. 3:**
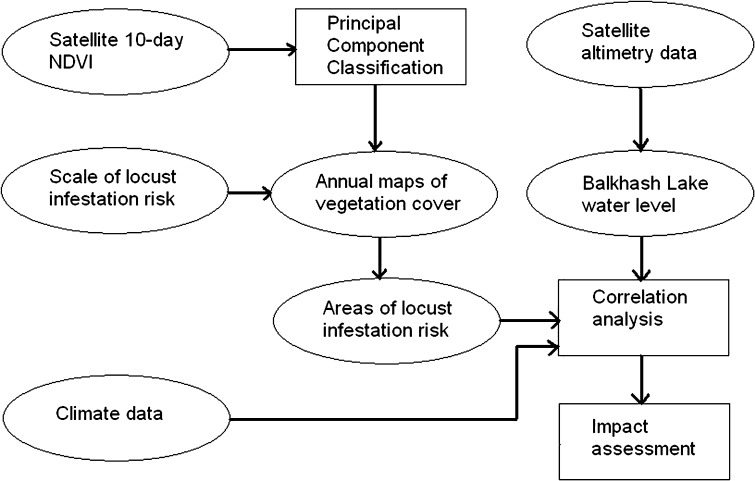
Monitoring system for locust infestation risk

Figure 3 illustrates the generalized processing stream and data involved in the monitoring system. The ellipsoids represent monitoring system inputs, whereas the boxes symbolize analysis steps. Detailed description of the data and individual processing steps presented in the flowchart follow in subsections vegetation-cover classification, assessing the risk of locust infestations for vegetation classes, and analysing the impact of environmental factors on risk of locust infestations.

### Vegetation-Cover Classification

A vegetation cover–classification scheme was developed consisting of seven thematic classes: solonchaks, sparsely vegetated, flooded reeds, vegetated sands, grassland, Tugay forests, and reeds (see also Table 1), and each related to an element of locust infestation risk. Analysing plant growth cycle is very informative when attempting to identify different vegetation types using remote-sensing data. Plant species found in forests, wetland, rangeland, etc., have different seasonal cycles (Yool and others 1997; Wang and others 2001).

**Table 1:** Risk of locust infestation for different vegetation classes in the Balkhash Lake basin

Vegetation class	Risk of locust infestation		
	Low	Medium	High
Solonchaks	+		
Sparsely vegetated	+		
Flooded reeds	+		
Sand dunes	+		
Grassland		+	
Tugay forests		+	
Reeds			+

**Fig. 4:**
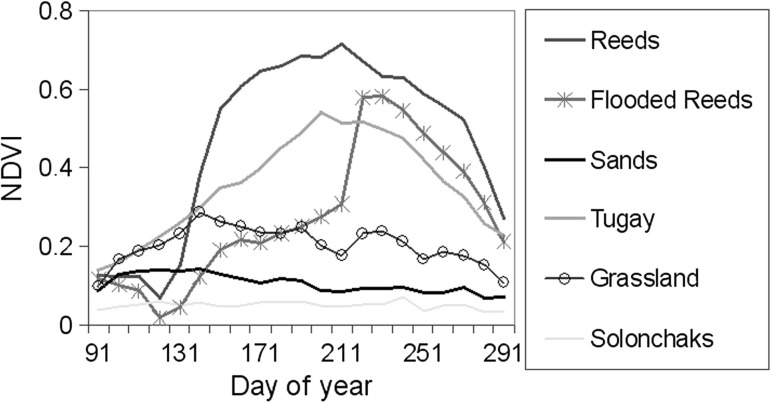
NDVI data from April 2003 through October 2003 obtained at five locations in different vegetation classes in the study area. Each value represents the average of nine pixels (approximately 9 km²)

In our vegetation-classification scheme, the distinguishing of the classes was based on an analysis of multitemporal dynamics of SPOT-VGT NDVI values observed by individual vegetation classes. Each of the vegetation types in the study area has a characteristic pattern of NDVI variation during the growing season, which can be used as a “foot-print” for the identification of pixels associated with a certain vegetation class (Fig. 4). Distinguishing between different individual vegetation types was performed based on NDVI profile analyses.

The seasonal changes in NDVI observed by the vegetation categories provided an opportunity to classify the vegetation cover (Tucker and others 1985). A principal-component analysis (PCA) procedure was used for 21 different 10-day NDVI periods between April and October in each of the growing seasons from 1998 through 2007. In the PCA, the NDVI was the variable used from time 1 to time 21 (number of 10-day periods within the growing season). The *n* PCs are related to the original variables by the equation:

2 $$ P_{i} = \sum {a_{ij} {\text{NDVI}}_{j} } $$

where $a_{ij}$ are the eigenvectors ranked in order corresponding to the size of the eigenvalues and NDVI_*j*_ is an NDVI value for time *j*. Each eigenvector represents the relative contribution of each time period to the PC and is obtained by solving the equation:

3 $$ C_{{\lambda_{ij} }} = a_{ij} \lambda_{ij} $$

where C is the covariance matrix of the NDVI for the 21 different 10-day periods, and $\lambda_{ij}$ is the positive eigenvalue representing the variance of each PC. The first eight PCs were generated for each growing season. The first PC produced by this technique has been reported to reflect the general spatial pattern of vegetation cover, whereas the second PC represents vegetation-type seasonality (Tucker and others 1985). For this study, the first and second PCs were used as classification inputs to the parallelepiped approach (Jensen 1996).

Classification procedure was performed using ENVI 4.8 image analysis software (ITT Corp). We selected areas of known vegetation types to be used as a classification-training area based on the field data and agreement among various cartographic data sets. These training areas, which represented small percentage of the total area of the Balkhash Lake basin, were used to label the feature space formed between the first and second PCs for each pixel of the study area. Boundaries of the various vegetation types were determined on the basis of the location of the training areas in the feature space and the classification image produced. The mapping process was repeated for every year from 1998 through 2007.

Accuracy of the classification procedure was examined using geo-referenced ground-collected data, comprising ground truth points, topographic maps, and fine-resolution satellite images (see Section Ground Truth and Verification Data). The Kappa coefficient was used as a guide to assess classification accuracy.

### Assessing the Risk of Locust Infestations for Vegetation Classes

We used a three-category (low, medium, and high) scale to assess vegetation areas with respect to their susceptibility to be infected by locust swarms (Table 1). Susceptibility of individual vegetation classes to locust infestation risk was taken from previous works in the region (Sivanpillai and others 2006; Kambulin 1992). The highest risk was associated with reed stands. Such habitats provide favourable conditions for locust feeding and egg-laying. Vegetation complexes in river valleys (Tugay forests), where reeds are mixed with trees and grasses, were assigned a medium risk. Medium-risk habitats included grasses, forbs, and shrubs (vegetation class “grassland”). Finally, solonchaks (strongly salinized areas), reed stands submerged in water (flooded reeds), and sand dunes represent the habitats with low risk of locust infestation.

### Analysing the Impact of Environmental Factors on Risk of Locust Infestations

To examine influence of climatic and hydrological factors on risk of locust infestations in the study area, we analysed the spatial extent of potential habitats of L. *migratoria migratoria* for its response to climate dynamics. The first part of the infestation dynamics assessment was quantified with correlation analysis between different degrees of locust infestation risk in individual years and climatic variables. We also examined the association between the hydrological regimen of the Balkhash Lake and locust risk. In this analysis, the spatial extent of potential locust habitats was compared with the water level in the Balkhash Lake obtained from the altimetry data.

Statistical significance and strength of the associations between the environmental factors and spatial extent of the potential locust habitats was tested by Student *t* test. The criterion for significance was set to *P* < 0.05. Finally, multivariable three-dimensional diagrams were generated to summarize and examine the evolution of the areas associated with locust habitats during the period from 1998 through 2007.

## Results

### Growing-Season Dynamics of NDVI in Individual Vegetation Classes

The data depicted in Fig. 4 show growing season profiles of NDVI for five of the seven individual vegetation classes from the study area. All presented vegetation classes demonstrate considerable variation in seasonal NDVI dynamics. However, the amplitude and patterns of these variations differ between the vegetation classes. Vegetation-free solonchaks were the only area observed to be temporally invariant. There are also differences between vegetation types regarding greening-up and peaking time. Sand dunes and grassland have their growth peak at the end of May to the beginning of June, whereas other vegetation types show the greatest growth significantly later at the end of June to the beginning of July.

Thus, the NDVI curves of Tugay forests show profiles with only one sharp peak at the middle of July, whereas reed grass exhibit a more rounded profile with a prolonged period of the greatest growth (from the beginning of June through the end of July). Tugay forests are associated with distinctly greater NDVI values in the beginning of the growing season (April through May) than the other presented vegetation types. The temporal NDVI profile of reeds submerged in water is similar to that of reeds only during the third part of the growing season (the end of July through October), when the flooding time is finished, and the largest part of reed plants stays above the remaining water. During the first and second parts of the growing season, the flooded reed plants are characterized by low NDVI values because most of the plant is submerged in water.

### Classification of Vegetation Cover and Areas of Locust Infestation Risk

As a result of the vegetation-cover classification (section Vegetation-Cover Classification), we obtained 10 annual distribution maps for the vegetation classes. Accuracy assessment was conducted using ground reference data points for 2004 through 2007. It produced an overall accuracy of 76 % and an overall Kappa coefficient of 0.719. Most of the errors identified in the accuracy matrix were due to misclassification of the following classes: reeds/flooded reeds and Tugay forests/reeds. It was evident that a certain proportion of reference points corresponding to reeds were misclassified as flooded reeds or Tugay forests Table 2.

**Table 2:** Satellite-based estimation of areas for various vegetation-cover classes (km²) averaged from 1998–2007

Vegetation type	Risk of locust infestation	Area	
		Km²	% of total
Solonchaks	Low	1,679	1.82
Sands	Low	67,099	72.96
Grassland	Medium	9,266	10.07
Tugays	Medium	6,848	7.44
Reeds	High	5,682	6.17
Flooded reeds	Low	1,383	1.50
Total		9,1957	100

**Fig. 5:**
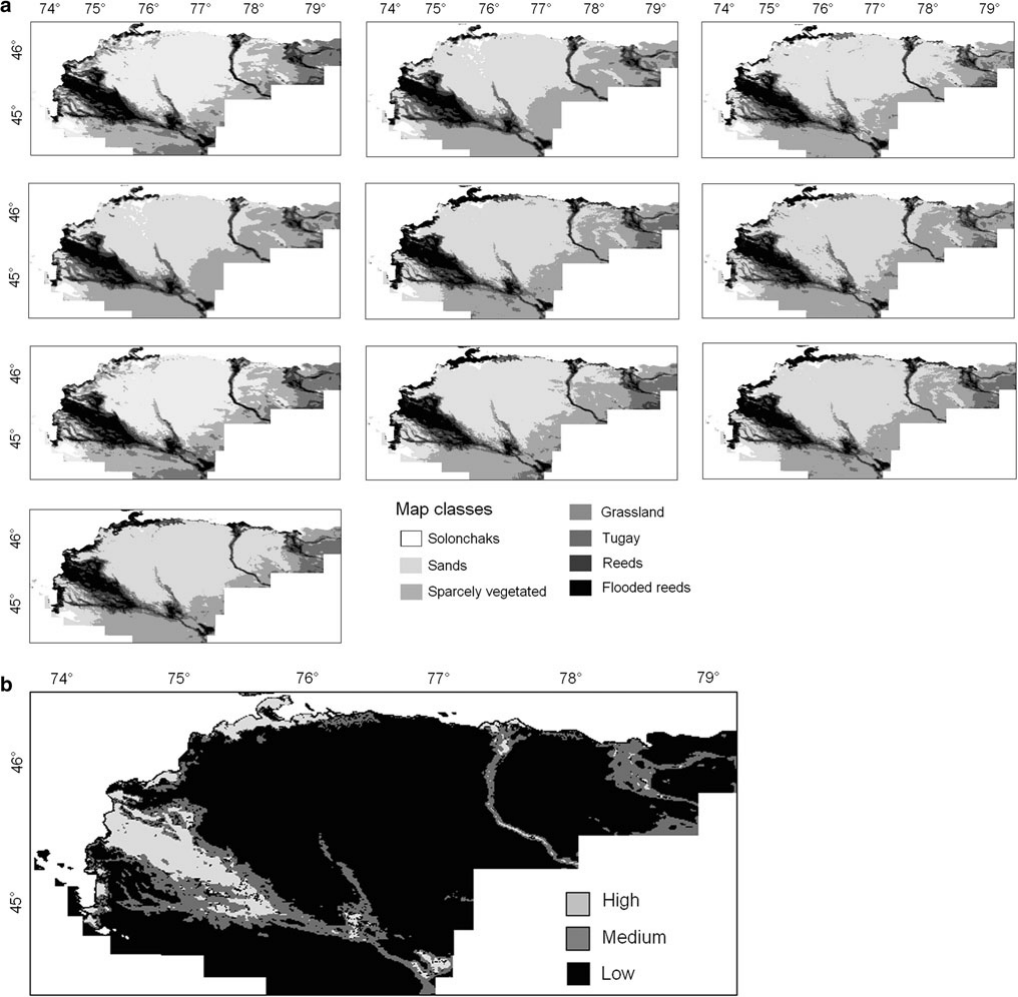
Annual maps of distribution of vegetation classes (**a**) and areas of potential risk of locust infestation in the Balkhash Lake drainage basin averaged for the entire period (**b**)

Area estimates obtained from the vegetation-cover maps from 1998 through 2007 indicated that, averaged from 1998 through 2007 (Fig. 5a), 6.17 % of the Balkhash Lake drainage basin was occupied by reeds (Table 3). These areas represent a high risk in terms of potential locust habitats. In addition, approximately 7.5 % of the region was occupied by Tugay vegetation in river valleys where reeds are mixed with other vegetation. These areas represent medium risk in terms of potential locust habitats.

**Table 3:** Nonlinear *R*² between spatial extensions of areas associated with high to medium risk for locust infestation and environmental variables using third-order polynomial regression

Vegetation type	Risk of locust infestation	*R*²					
		Temperature		Precipitation		Water level	
		No lag	1 lag	No lag	1 lag	No lag	1 lag
Tugay forest	Medium	0.37	0.65	0.28	0.10*	0.78	0.34
Reeds	High	0.53	0.54	0.26	0.38	0.89	0.56
Tugay + reeds	High to medium	0.43	0.67	0.11*	0.33	0.88	0.44

* The determination coefficient is not significant at the level of *P* < 0.05

Together, the areas of high and medium risk represented >13.5 % of the Balkhash Lake drainage basin, which should become a focus for locust population monitoring and management activities. Pixels classified as grassland vegetation constituted 10.07 % of the study region area. They were identified as medium to low risk for potential locust habitats.

Reeds submerged in water were termed low risk for potential locust habitats and occupied 1.5 % of the total area. Other vegetation classes, such as solonchaks and vegetation of sands, occupy 74 % of the study region and represent low risk for locust infestation. Figure 5b depicts the average vegetation class distribution as it relates to potential locust infestation risk for 1998 through 2007.

### Dynamics of Locust Habitat Extension and Environmental Factors

**Fig. 6:**
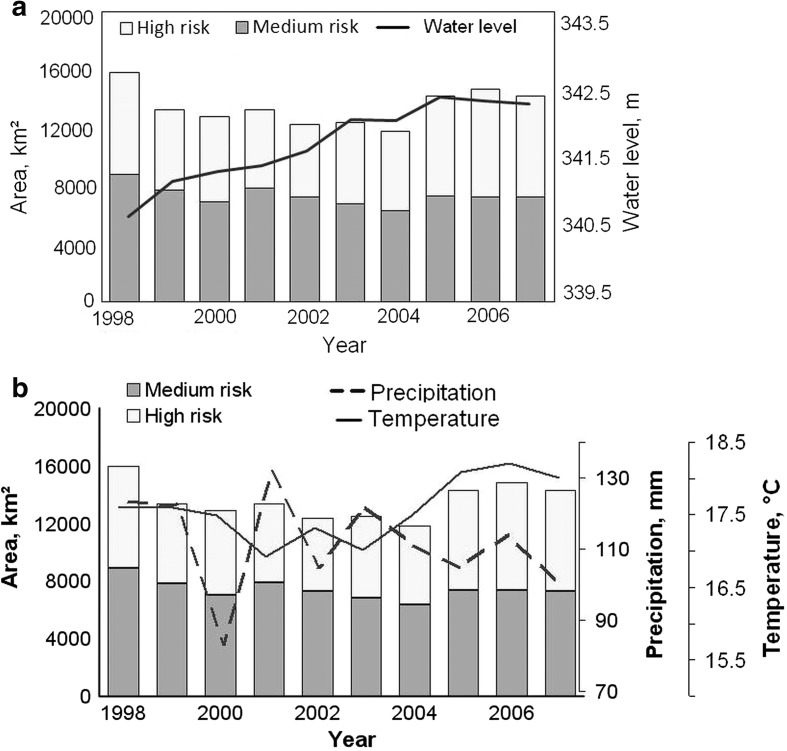
Extension of areas associated with high and medium risk for locust infestation plotted *versus* the water level of Balkhash Lake from 1998 through 2007 (**a**). Dynamics of growing season temperature and precipitation at the Saryshagan climate station from 1998 through 2007 (**b**)

The classification results define interannual variability of areas occupied by the individual vegetation classes. For our study, changes of areas associated with high and medium risk for locust infestation were of particular interest. The annual dynamics of these areas from 1998 to 2007 are presented in Fig. 6. Consider the 1-year time lag of the precipitation/temperature graph. A visual analysis of the diagrams in Fig. 6a, b gives an impression that there is strong association between extension of the areas and growing season temperature: Increasing temperature enforces an increase of the areas associated with high to medium risk of locust infestation.

Years with particularly high temperature and low precipitation (1997, 2000, and 2005) preceded years with the greatest risk of potential locust habitats (1998, 2001, and 2006). There also is a relationship between the water level of Balkhash Lake and the areas (Fig. 6a). However, this relationship is not as obvious as in the case of temperature. Indeed, during 1998 through 2002, an increase in water level was not accompanied by an increase of areas. In contradiction, the area of potential locust habitat temporarily decreased from 1998 through 2002. During the second part of the study period (2003 through 2007), the area of high to medium risk seems to be proportional to the water level of Balkhash Lake. We suggest that the relationship between water level and area of potential locust habitat is nonlinear. Therefore, in this case linear correlation analysis would be confusing.

### Relation Between Environmental Factors and Risk for Locust Infestation

To quantify the relationship between locust infestation risk area and environmental factors, we calculated the coefficient of determination (*R*²) between variables performing bivariate nonlinear regression analysis (third-order polynomial). The analysis was performed on a concurrent basis using coherent time series of the variables, with a single-year time lag when inputted into the statistical regression. The results of the regression analysis are listed in Table 3. Indeed, temperature correlates with each of the vegetation types; however, we should take into account two different impacts of temperature on vegetation growth.

For analysis at the concurrent basis, the results, as might be expected, indicate that temperature is one of the major climatic factors enforcing plant growth in this area: Above a certain base value, a plant’s growth rate is proportional to temperature. For Tugay, reeds, and Tugay + reeds, the results demonstrate that influence of temperature from the preceding year is stronger than from the concurrent year. In this case, we must assume that the impact of temperature is indirect: Temperature influences other variables that have a direct impact on vegetation growth during the concurrent year.

Recent literature reported about the consistency of temperature with the hydrological regime of the Balkhash Lake’s contributing rivers (Tlenbekov & Piven 1993). Dry years with high temperature cause a significant decrease in rainfall and snow accumulation in the Balkhash Lake drainage basin, which, as a consequence, results in a decrease of run-off during the following year. From that reason, larger areas are free from seasonal flooding in the deltas of the Ili River and other rivers, thus expanding the habitat suitable for locust oviposition and subsequent nymphal development during the next year. These results support the previous observation by visual analysis of Fig. 6.

The statistically significant *R*² values for the analysis with one lag show the general impact of the preceding year’s precipitation on the subsequent year. Nonetheless, the influence of rainfall is much weaker than that of temperature. The results show that in the analysed vegetation categories, the major source of water available for plant growth is surface water from periodic flooding. These vegetation types show strong correlations with the water level of Balkhash Lake. The dynamics of the water level explain >80 % of the variance in reed areas. For Tugay forest, the explanatory power is somewhat lower (*R*² = 0.78). The results associated with Tugay and reeds suggest that precipitation also plays a role in plant growth of these vegetation classes, even although this role is small.

## Discussion

**Fig. 7:**
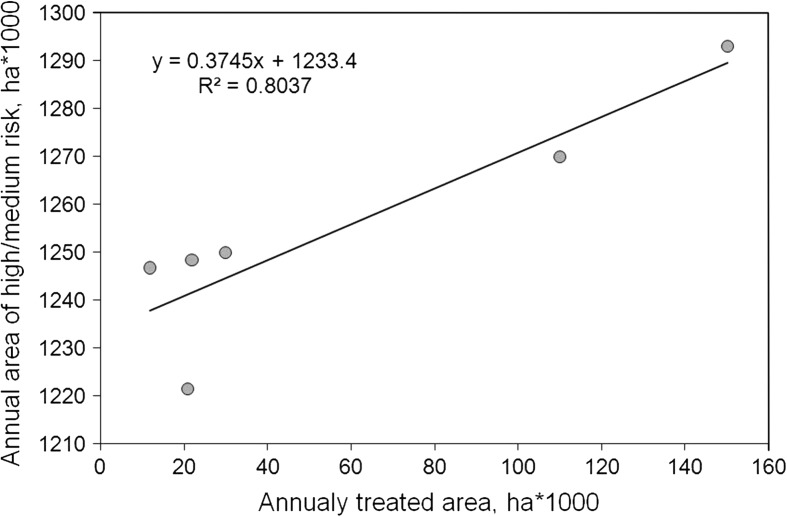
Comparison between annual potential locust infestation areas in the Balkhash Lake basin and areas annually treated against locusts in the Ili River delta for 1998 through 2003. The data on the annually treated area was taken from Sivanpillai and others (2006)

In the study region, the vegetation exhibits strong and predictable seasonality, whereas seasonal profiles of various vegetation types differ strongly from each other. The use of coarse spatial resolution SPOT-VGT data from different years enabled us to capture yearly variations in vegetation cover caused by climatic dynamics. The classification approach, combined with PCA, enabled us to construct vegetation-cover maps based on the multitemporal responses of vegetation classes. There was a good qualitative agreement with the distribution of vegetation observed in field and in a set of topographic maps and field survey data. The significance of the classification results rests also on the comparison of locust outbreaks relative to the interannual change in the potential locust infestation areas (high and medium risk). These areas correspond well to the annual areas of insecticide treatments (Fig. 7).

It was found that averaged over the study period from 1998 through 2007, only approximately 13.5 % of the total area of the Balkhash Lake basin was subject to locust infestation. Approximately 10 % of the total area was assigned a medium to low risk of locust infestation. The remaining area represented habitats with low risk of locust infestation. The present study used similar classes related to the risk of potential locust infestation similar as Sivanpillai and others (2006) for the Ili River delta. However, the percentages of areas with risk of locust infestation obtained in our study were incomparable with those reported by Sivanpillai and others (2006) because the study area of the presented investigation was approximately 10 times greater than the Ili River delta (Sivanpillai and others 2006).

**Fig. 8:**
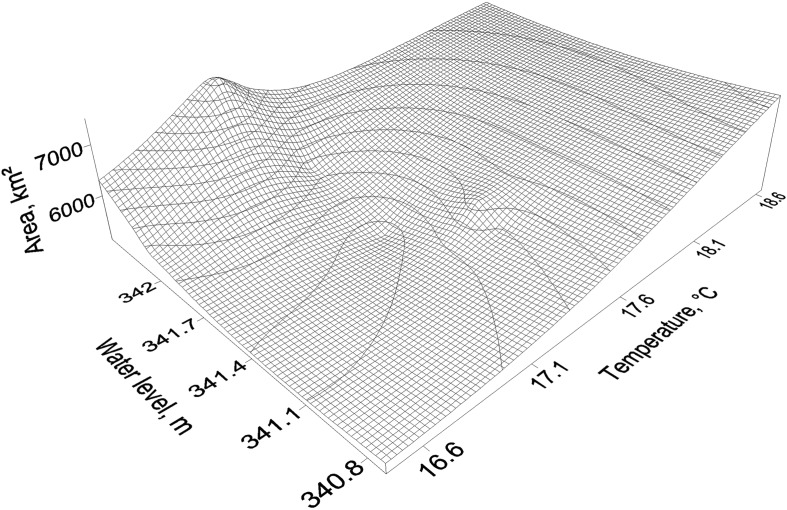
Area of potential locust habitats in a 3D space of temperature and water level in Balkhash Lake

The breeding habitats of the Asian migratory locust are linked to natural thickets of reed, which provides a source of food. Such habitats, together with areas of medium risk, cover >13 % of the territory in the Balkhash Lake drainage basin. The results of this study show that periodic fluctuations of the water level in the lake influence the total area of the potential locust habitat and, as a consequence, locust population dynamics: When the water level decreases, the area for locust breeding increases, and vice versa (Fig. 8). Therefore, high water levels in Lake Balkhash is one of the major issues regarding locust control and prevention of locust plagues in the region.

Because the area of potential locust habitats correlates positively with temperature, ongoing global climate change prompts locust hazard risk. However, the issue of contemporary warming has only been infrequently discussed in the content of climate impact on locust vulnerability. The international society and policy-makers should also recognize this issue of climate change. At the scale of the Balkhash Lake region, climate conditions should be extensively monitored regarding forecast outbreaks of Asian migratory locusts. The results of this study show that temperature and precipitation can be used as effective indicators of locust risk. Higher temperature and lower precipitation amounts, in combination with a lower water level in Balkhash Lake, are reliable signs for high risk of a locust outbreak.

However, the network of meteorological stations in the region of Balkhash Lake is not sufficiently dense, and the quality of climate observations at stations shows many deficiencies in measurements and reports. The constitutional change had a negative influence on the collection of climate data in Kazakhstan as a whole and in the region of Balkhash Lake in particular. After the collapse of the Soviet Union, a number of climate stations in the region were abandoned because of diminished financial support. Similar problems are found in the observation of Lake Balkhash water levels. Regular lake-level gauge measurements are practically absent from 1993 to the present. In this sense, satellite-altimetry observations provide a unique opportunity to establish a monitoring system of the water levels in Balkhash Lake.

Obviously, the locust control system was negatively affected by the transformation of the political system in Kazakhstan during the 1990 to 2000 s. The new socioeconomic system gave/gives low priority to meteorological and hydrological observations as well as plant protection service, which are indispensable for effective locust control. Once locusts infected huge areas in southern and northern Kazakhstan and destroyed a large part of cereal crop yield during the locust plague from 1998 to 2001, decision makers realized that the locust-control system requires strong support by the state as well as collective action (Latchininsky and others 2007; Toleubayev and others 2007). However, efforts to establish an ecological view of locust control, as well as the ability to understand relationships between climate, hydrological regime, land-use change, and locust hazard risk, are incomplete.

## Conclusion

This study investigated the link between environmental conditions and areas of potential locust infestation. For this purpose, the 10-day time series of the NDVI, derived from SPOT-VGT satellite, was used for mapping potential locust habitats and monitoring their area during the period from 1998 though 2007. Environmental conditions were represented by 10-day climatic data from a climate station located in the study area, and the water level in Balkhash Lake was derived from satellite altimetry observations (T/P/Jason 1).

The results demonstrate that SPOT-VGT data are a powerful tool for mapping areas threatened by locust hazard and monitoring their interannual dynamics over large regions. Because of low precipitation and air humidity in the study region, most of the used SPOT-VGT composites were cloud-free. Existing noisy composites in the data set were corrected using a spatio-temporal filtering procedure (Chen and others 2004) that calculated new values for the noisy pixels using their spatial and temporal neighbours. This data-preprocessing step may be one of the reasons for some resulting misclassification. Certainly, future progress in this research field will improve the accuracy of classification.

The presented combination of SPOT-VGT data with altimetry data from T/P/Jason 1 promises great potential for establishing a low-cost and effective multisensor monitoring system for mapping locust habitats in the Lake Balkhash drainage basin. Moreover, because areas for locust breeding are closely linked to the water level and temperature of a previous year, forecasting locust hazard risk is absolutely possible.
